# Desktop Virtual Reality Offers a Novel Approach to Minimize Pain and Anxiety during Burn Wound Cleaning/Debridement in Infants and Young Children: A Randomized Crossover Pilot Study

**DOI:** 10.3390/jcm12154985

**Published:** 2023-07-28

**Authors:** Taima Alrimy, Wadee Alhalabi, Areej Malibari, Fatma Alzahrani, Sharifah Alrajhi, Ayman Yamani, Halah Ahmed, Amro Abduljawad, Essam Nasser, Samar ALattar, Buthinah Alharby, Hasna Khalid, Mohammed Alhalabi, Hunter G. Hoffman, Keira P. Mason

**Affiliations:** 1 Computer Science Department, Faculty of Computing and Information Technology, King Abdulaziz University, Jeddah 21589, Saudi Arabia; 2 Immersive Virtual Reality Research Group, King Abdulaziz University, Jeddah 21589, Saudi Arabia; 3 Paediatric Department, King Abdulaziz University, Jeddah 21589, Saudi Arabia; 4 Statistics Department, Faculty of Science, King Abdulaziz University, Jeddah 21589, Saudi Arabia; 5 Department of Plastic Surgery and Burn, Alnoor Specialist Hospital, Makka 24241, Saudi Arabia; 6 Burn Unit, King Abdulaziz Hospital, Jeddah 22421, Saudi Arabia; 7 Plastic Surgery Department, International Medical Center, Jeddah 23214, Saudi Arabia; 8 Department of Mechanical Engineering HPL, University of Washington, Seattle, WA 98195, USA; 9 Department of Anesthesiology, Critical Care and Pain Medicine, Harvard Medical School, Boston Children’s Hospital, Boston, MA 02115, USA

**Keywords:** analgesia, pain, pain management, pediatric, virtual reality

## Abstract

Although most scald burn injuries involve children under six, because of the challenges of using head mounted displays with young children there is very little research exploring the use of VR in children under six. The current clinical pilot study measured the analgesic effectiveness of our new desktop VR system (with no VR helmet) in children under six during burn wound care (a within-subjects design with randomized treatment order). Between December 2021–April 2022, nine children with burn injuries (10 months to 5 years age, mean = 18 months) participated. The mean burn size was 10% Total Body Surface Area, range 2–22%. Using nurse’s ratings, VR significantly reduced children’s pain during burn wound care by 40% on the observational Faces, Legs, Activity, Crying, and Consolability (FLACC) pain scale. Specifically, non-parametric within-subject sign tests compared nurse’s ratings of the young patients’ pain during burn wound care using usual pain medications with no VR = 6.67, (SD = 2.45) vs. adjunctive Animal Rescue World VR (VR = 4.00, SD = 2.24, *p* < 0.01). The observational Procedure–Behavior Checklist (PBCL) nurse’s scale measured a 34% reduction in anxiety with VR as compared to pharmacologic treatment alone (*p* < 0.005). Similarly, when using single graphic rating scales the patients’ parents reported a significant 36% decrease in their child’s pain during VR (*p* < 0.05), a 38% (*p* < 0.005) decrease in their child’s anxiety during VR, and a significant increase in patients’ joy during VR. It can be concluded that during burn wound care with no distraction (traditional pain medications), children under 6 years old experienced severe pain during a 10 min burn wound cleaning session. During burn wound care combining desktop virtual reality and traditional pain medications, the same pediatric patients experienced only mild pain during burn wound cleaning/debridement. VR significantly reduced the children’s pain and anxiety during burn wound care.

## 1. Introduction

The treatment of burn injuries in the pediatric population is challenging. Although a wide variety of pharmacologic therapies have been trialed and implemented, children continue to suffer during burn dressing changes and continue to be exposed to risks and side effects associated with sedation, analgesia, and anesthesia [1,2,3,4]. Even when protocols are adopted to optimize analgesia and anxiolysis during dressing changes, the protocols are often not implemented in actual practice [5]. As children suffer during burn treatments, parents experience symptoms of anxiety, guilt, and depression, which can affect the child’s response to therapeutic interventions [6]. 

A growing body of literature suggests that repeated exposure to severe and excruciating pain during frequent burn wound care sessions may lead to pathological neural plasticity changes in a child’s brain, leading to unhealthy persistent changes in their pain perception system, (chronic pain/central sensitization), and/or post-traumatic stress disorder (PTSD) symptoms [7,8,9,10]. 

Opioids remain a mainstay for analgesia during burn dressing changes, despite concern that opiate exposure, albeit for medical therapy, can lead to opiate abuse [11]. A survey of American Burn Association centers revealed that although opioids were prescribed by 100% of survey respondents, up to 50% of children did not have adequate analgesia and anxiolysis with the initial sedation regimen [4]. Concerns about repeated exposure to sedatives and pain medications has led to a Food and Drug Administration (FDA) warning “that repeated or lengthy use of general anesthetic and sedation drugs during surgeries or procedures in children younger than 3 years or in pregnant women during their third trimester may affect the development of children’s brains” (FDA, 2017 p. 1) [12]. Anesthesia and sedation have been associated with short-term behavioral and emotional change, decreased academic function, an increase in unhealthy behaviors (eating and sleeping difficulties, withdrawal, apathy, enuresis), and a fear of future medical visits [13,14,15,16,17,18,19].

Over the past few years there has been a growing interest in the application of non-pharmacologic techniques to be used either instead of or as an adjunct to sedation [20,21,22,23,24]. Most relevant to the current study, non-pharmacologic techniques have been shown to be effective for children under six years old. For example, “in person” support of patients by a child life specialist [25] and entertainment by live clowns [26] have been shown to help reduce pain and anxiety of young children under six during burn wound care. In light of the current opioid overdose crisis, new federal regulations are greatly reducing the availability of opioid analgesics, while at the same time there is growing pressure to improve pain management in light of growing awareness that undermedication can have expensive long-term consequences for patient health. A new technique that can reduce pain during medical procedures and simultaneously reduce reliance on opioids is needed. Development of more powerful new non-pharmacologic analgesics has become a national priority [20].

The first evidence that immersive Virtual Reality (VR) can reduce procedural pain and anxiety appeared in the late 1990s [27,28] using a very rare early VR system with an 8 lb VR helmet. Recently, with the improving technology associated with artificial intelligence and virtual reality, these techniques are increasingly being applied as distraction methods in both pediatric and adult patients [29,30,31,32,33]. Virtual reality is defined as “an artificial environment which is experienced through sensory stimuli (such as sights and sounds) provided by a computer and in which one’s actions partially determine what happens in the environment” [34]. There is growing evidence that virtual reality can serve as such a powerful distraction that it may be capable of reducing acute pain when applied during painful medical procedures.

VR delivers an interactive computer-generated experience that immerses the child in the illusion of being inside a 3D computer generated world, as if the virtual world is a place they are visiting. When immersed in VR, the user has less attention available to process incoming signals from pain receptors, offering hope that VR may provide non-pharmacologic analgesia [35,36]. Patients have reported less pain while in VR, spend less time thinking about their pain during VR, and often report having more fun during wound care while in VR compared to wound care with no VR [28,37,38,39,40]. According to fMRI studies, VR significantly reduces pain-related brain activity [41]. An fMRI laboratory study comparing VR analgesia to opioid analgesia in healthy adults showed that VR reduces subjective pain as much as a moderate dose of hydromorphone [42]. Similarly, VR alone and opioids alone showed similar reductions in pain-related brain activity [42]. Despite the growing evidence that VR can reduce pain in pediatric burn patients over 6 years of age [32,39,43] there is limited research on its success and applicability in infants and younger children under six. As a rare exception, a group of researchers in Montreal have recently explored the use of projector-based VR for young children during burn wound care by using observer ratings of patient’s observable pain behavior [44]. In these studies, young children interacted with VR images projected onto an immersive dome rear-projection screen positioned near the patient; it was found that projector-based VR reduced the pain of young children during burn wound care with no need for a VR helmets [45], which is encouraging for further research.

Finding adjunctive therapies and evaluating the use of VR in this younger age group is of critical importance, as 61% of all scald burns (including adults) occur in children under 5 years old [46].

### 1.1. Desktop Virtual Reality System Design

The current study is the final stage of a multi-stage process. An online survey of parents and guardians of children aged 0 to 5 years old was conducted by Taima Alrimy. This survey was designed to determine what kinds of technology young children were currently using (if any), and especially what types of VR experiences children would likely prefer. The survey results indicated that children were familiar with TV screens (i.e., desktop VR type displays) and that children loved cartoons, animals, and sounds. The design of the VR system used in the current study, called Animal Rescue World, took the children’s early stage of development and limited language and cognitive ability into consideration. Large, simple, and easy to identify virtual objects were used, and the children’s interactions with the virtual objects in Animal Rescue World were simple enough for young children to perform, anticipating that they would be having their burn wounds cleaned and would likely be in pain during VR. VR Animal Rescue World is designed to be simple and easy to use for young children. Attractive colors, sounds, and popular animated animals were used. The user floats slowly along a path in an open field in the virtual outdoors. Familiar domestic and wild animals float in the air, trapped in large bubbles (e.g., a rabbit, an eagle, a kitten, a tiger, an elephant, etc., one animal per large transparent bubble). Each animal makes sounds indicating whether they want out of the bubble. The child “frees” the imprisoned animals by using the computer mouse to touch the bubble with their cursor and click the bubble to burst it, freeing the animal. The animal then floats slowly to the ground of the African savannah and makes sounds indicating that it is happy to be free and on the ground. When on the ground, if the patient touches the animal with the cursor after freeing the animal, the animal glows briefly. Then, the patient slowly floats to the next bubble, and as the pediatric patient frees one animal after another their viewpoint slowly moves along the path in the virtual African Savannah. An interactivity score is displayed showing the user’s interaction success.

### 1.2. Software and Materials

**Figure 1:**
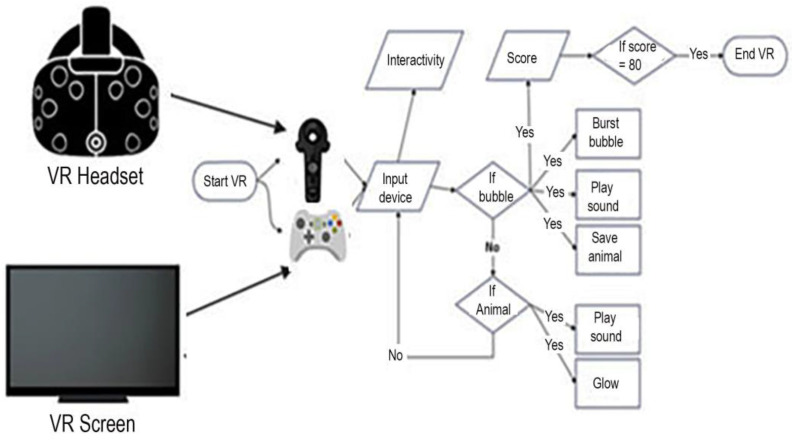
Virtual reality flow chart.

The VR world was built using the Unity^®^ game engine and Visual Studio for coding in the object-oriented C# programming language. The game computer we used was a Windows^®^ 10 HP gaming laptop powered by a 2.80 GHz Intel^®^ Core i7-7700HQ CPU, 16 GB of RAM, and an NVIDIA^®^ GeForce R GTX 1050 Ti GPU with up to 8 GB of dedicated video memory graphics. As shown in Figure 1, the software can either be used with an immersive VR system using an HTC VIVE head mounted display with head tracking and hand controllers, or it can be used as Desktop VR, using a computer mouse-type game controller input device to allow the child patient to interact with the bubbles and virtual animals. Only desktop VR was used in the current study. The VR experience begins as soon as the experimenter hits the start button, and the patient’s viewpoint floats slowly along a pre-determined spline path. Each mouse click button pressed by the patient is counted as interactivity. Each time a bubble is clicked, the bubble disappears with a sound, the animal inside floats down to the meadow, and the score is updated. 

**Figure 2:**
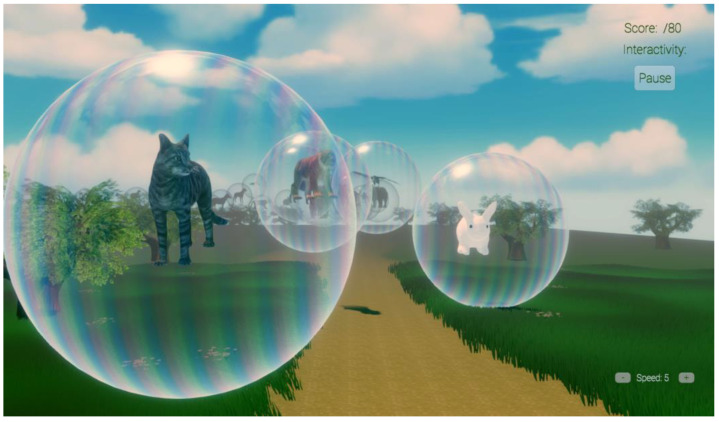
A screenshot of Animal Rescue World. Image by Taima Alrimy, copyright Hunter Hoffman, www.vrpain.com (accessed on 27 October 2022).

**Figure 3:**
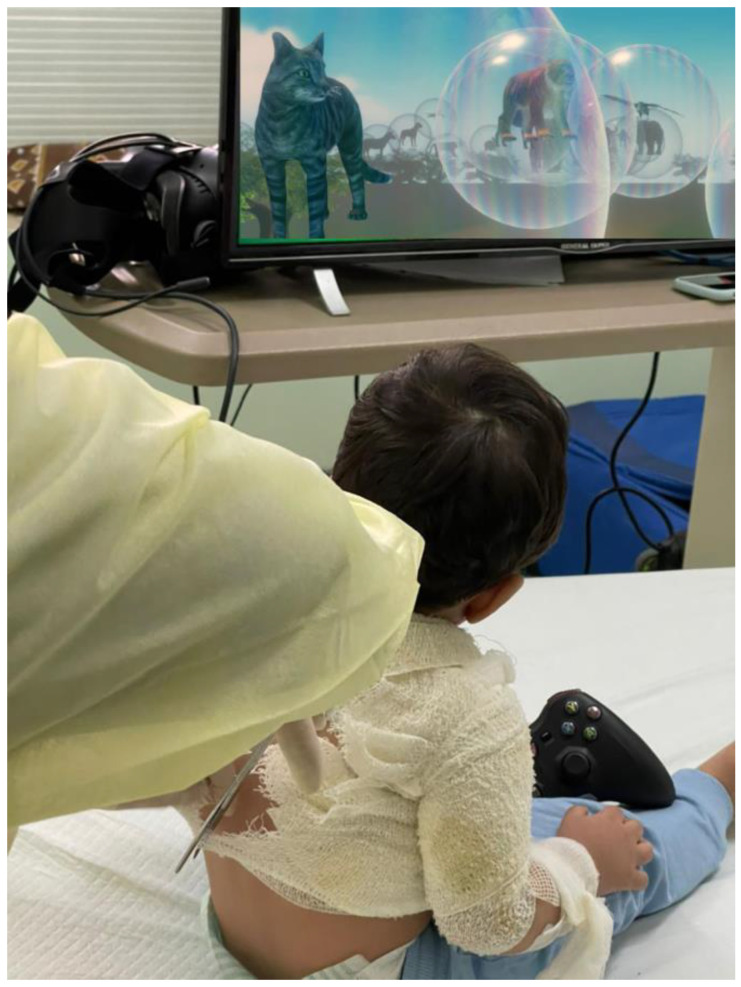
A young burn patient interacting with the Desktop Animal Rescue World during burn wound care. Photo by Taima Alrimy, copyright Hunter Hoffman, www.vrpain.com (accessed on 27 October 2022).

This study was the first to test the feasibility of using desktop VR during burn wound care in young children. In this study, young children interacted with creatures in a computer simulated environment using desktop VR. Although “desktop VR is less immersive than a head-mounted display (HMD) VR” (Liaw et al., 2022, p. 2, [47]; and see [48]), our custom interactive VR software is specifically designed to distract young children during burn wound care without requiring a VR helmet. The software used in the current exploratory pilot study, “VR Animal Rescue World”, (see Figure 2 and Figure 3), is a VR world simulation of an outdoor world including an African savannah with occasional trees, blue sky, and white clouds; it was custom designed and programmed by Taima Alrimy for pain distraction of young children during painful burn wound care procedures. For the current study, we designed a 3D VR environment that is suitable for children aged 10 months and older during painful medical procedures. Because of the young age of the children, tolerating the application and proper fitting of VR headpieces would not have been practical or feasible. Although the computer architecture of our Animal Rescue software enables it to be used as part of a fully immersive VR system with a head tracked VR helmet and hand controllers (see Figure 1), we felt that the interactive desktop VR version with a television display (no VR helmet) would be better tolerated in this young child age group. 

The primary objective of the current study was to determine whether adjunctive desktop VR distraction (usual pain medications + interactive desktop virtual reality) could reduce pain during burn care/wound debridement compared to treatment as usual (usual pain medications + no virtual reality). The patient population was children under six with severe burns requiring burn wound care in a hospital (e.g., scald burns). Nurse’s ratings of patient pain behaviors during wound care with and without VR was the primary outcome measure.

## 2. Materials and Methods

Children with burns admitted to the burn unit in the hospital could participate. Parents’ written approval of child participation was obtained. Children were excluded from the study if they did not meet the eligibility criteria. 

Inclusion and Exclusion criteria. This study included children who were: (1) aged from 6 months to 5 years old (under six) suffering from a burn injury who were undergoing wound cleaning/debridement sessions in the hospital and (2) accompanied by a parent able to provide consent for participation. We excluded from the study children who: (1) were diagnosed with a disability that prevented them from interacting with the game, (2) tested positive for COVID-19, (3) had a diagnosis of epilepsy, or (4) who were allergic to opioids or other analgesics used for standard pharmacological treatment.

A within-subject crossover design was conducted. Each child experienced a VR treatment session during burn wound care on one day and traditional treatment (no VR) during comparable wound care on another study day. The treatment order was randomized.

This statistically powerful within-subject repeated measure design is frequently used in pilot studies [49]. Each child served as his/her own control, reducing noise variance, and received both VR (pain medication + desktop VR) and standard (pain medication + No VR) treatments during two equivalent wound care sessions of approximately 10 min on different study days, with the treatment order block randomized. Half of the patients received desktop VR on their first study day and no desktop VR on their second study day, while the other half received no desktop VR first and desktop VR second. 

A convenience sampling method was used to recruit participants upon their presentation to one of the following three hospitals: (1) Alnoor Specialist Hospital in Makkah, (2) King Abdulaziz Hospital, and (3) the International Medical Center (IMC) in Jeddah. Potential participants meeting the eligibility criteria were identified by the physical therapist, who informed the research nurse when they were scheduled for a burn wound care session. Parents of potential participants were then approached by the research nurse, who provided them with an explanation of the study and asked for their consent. Written informed consent was obtained from all parents of recruited children prior to the wound care session. Assent of the children could not be obtained in most cases, given the very young age of the participants. Parents were assured that if they did not want to participate in this study this would not impact the quality of their child’s traditional wound care and pain treatments. They were assured that agreeing to participate in the study would not prevent their child from receiving the standard pharmacological treatment and additional pain medication if needed.

Wound care consisted of wound cleaning, including tissue debridement (scrubbing off a layer of exfoliated dead skin cells from the burn wound). Although bandage removal can be painful, it was not included in this study, as bandages are usually removed prior to the main wound cleaning activities. For the purposes of this study no wound care was performed during the first 5 min, as this time was allotted for the child to become accustomed with the procedure’s setting. Patients then received comparable wound care lasting approximately 10 minutes per day on two different study days. 

The patients’ pain and anxiety were measured by the medical care providers (physicians and nurses) using the Faces, Legs, Activity, Crying, and Consolability (FLACC) scale, a behavioral/observational pain scale from 0 to 10 (0 = relaxed and comfortable, 7–10 = severe discomfort/pain) for children from 0 to 18 years old [50].

Nurse’s ratings of the child’s anxiety was measured using the Procedure Behavior CheckList (PBCL) scale, which comprises eight behaviors based on occurrence and intensity for a possible total score ranging from 0 to 40 [51]. 

Ratings were obtained from parents as well. Parents rated their children’s pain using the single question Wong–Baker Faces Pain Rating Scale, which shows a series of faces ranging from a happy face at 0, or “no hurt”, to a crying face at 10, which represents “hurts like the worst pain imaginable”. Parents answered a single question to rate their child’s anxiety during wound care on the Subjective Anxiety Scale, a series of faces ranging from 0 = relaxed to 4 = angry. Similarly, parents rated their child’s joy during the VR and traditional sessions (no VR) using the Joy Faces Scale, ranging from 0 = no joy to 5 = ecstatic, and briefly rated how satisfied they were with their child’s pain management during wound care.

A post-treatment survey was distributed to doctors, nurses, and parents involved in the study to assess the feasibility and acceptability of the intervention. The amount of time the patient spent in VR vs. no VR during burn wound care was recorded. All collected data from the experiment were analyzed using SPSS software to evaluate the effectiveness of the VR system in pain and anxiety distraction.

## 3. Results

**Figure 4:**
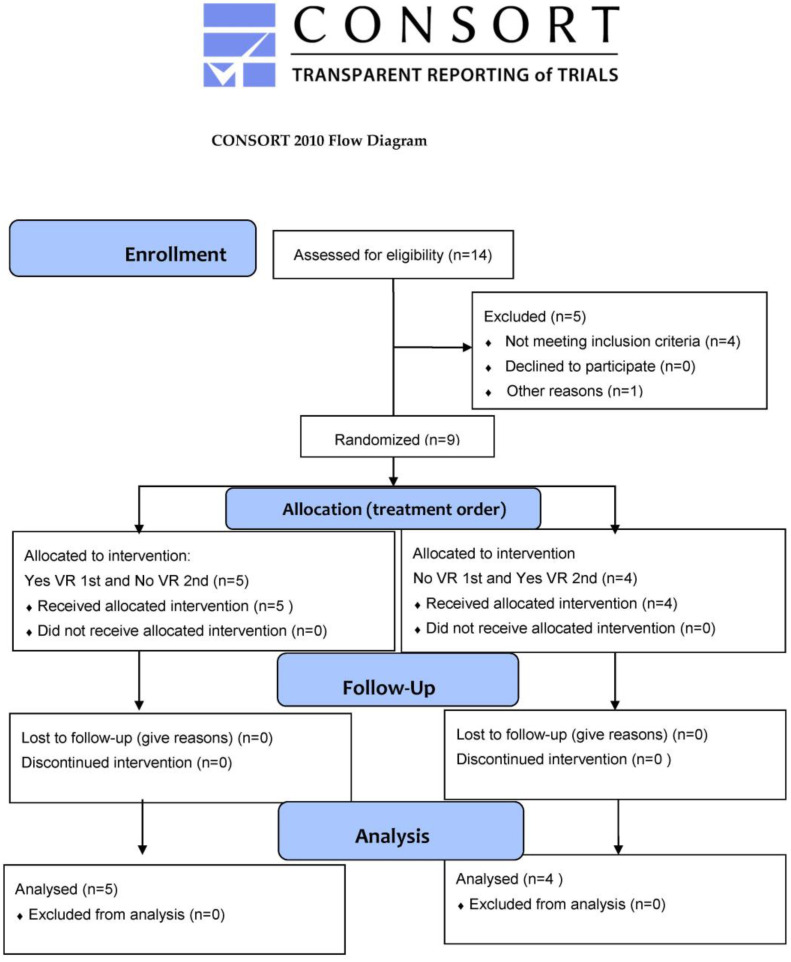
Consort flow diagram.

Fourteen children were recruited for the experiment by parents who signed both the informed consent and media release forms. Nine children completed wound care sessions on two study days, while five children were excluded, as illustrated in the Consort Flow Diagram below (Figure 4).

Table 1 shows the demographic characteristics of the participants; 67% of the children were female and 33% were male, while 89% were hospitalized in the burn unit and 11% were at the outpatient clinic. Most of the burns were caused by a hot substance, e.g., thermal liquids such as spilled hot beverages. All children took paracetamol in different forms (syrup, IV, pills, suppository) before wound care in addition to doses as background medication. 

Children were aged from 10 months to 5 years (under six); the mean age in months was (18.89 months), i.e., approximately one and a half years old. As for their weights, the lowest weight was (8 kg), the highest was (16 kg), and the mean weight was (10.31 kg). For the extent of the burn (burn size), the lowest value was 3% total body surface area (TBSA), while the highest value was 22% and the mean value was 10%. The lowest number of previous wound care sessions before participating in the current study was one and the highest was fifteen, with a mean of 4.3.

Table 2 below describes the injury/burn characteristics of the participants; 20% had deep partial thickness (second degree) burns, 30% had a superficial thickness (second degree) burns, 20% had both superficial and deep thickness (second degree) burns, 10% had both superficial (first degree) and superficial thickness (second degree) burns, and 10% had both superficial thickness (second degree) and full thickness (third degree) burns. The areas of burns were at different body parts for each patient, including the face, chest, anterior trunk, posterior trunk, limbs, hands, legs, thighs, and genitalia.

Observational scales of pain and anxiety results (Doctors’ and Nurses’ observations ratings of patient’s pain and anxiety) and total Faces, Legs, Activity, Crying, and Consolability (FLACC) scale pain scores were treated as ordinal data [52], as recommended and used by previous investigators [53]. Ordinal data require non-parametric statistics [54]. Furthermore, because we had a small sample size, determining the distribution of the primary outcome variable (the worst pain ratings during wound care) was important for choosing an appropriate statistical method. Normality is an important assumption of parametric statistics. Kolmogorov–Smirnov tests were performed, and showed that the distribution of the worst pain ratings departed significantly from normality for the worst pain during No VR, D(9)  =  0.332, *p*  =  0.005. Based on this outcome and the ordinal nature of the data, nonparametric statistics were used for all analyses.

**Figure 5:**
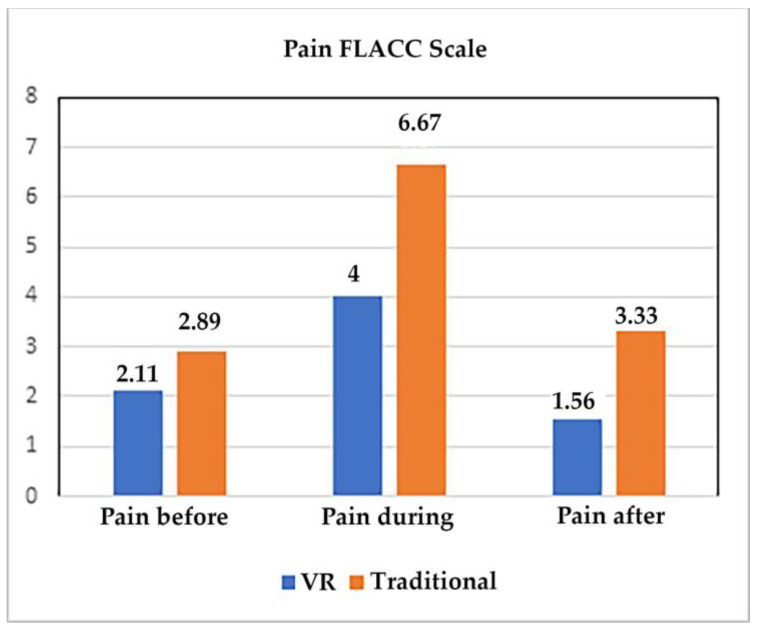
FLACC scale results (doctors’ and nurses’ ratings of patient pain before, during, and after burn wound care).

**Figure 6:**
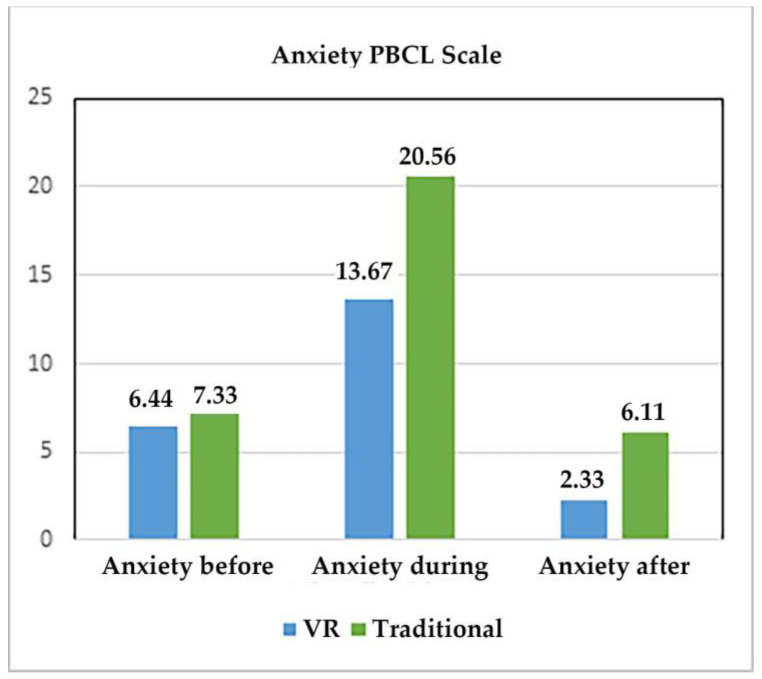
PBCL scale results (doctors’ and nurses’ ratings of patient anxiety before, during, and after burn wound care).

Table 3 shows the results of the nonparametric paired sign test used to measure the differences between VR and traditional treatment regarding pain before, during, and after burn wound care and anxiety before, during, and after wound care. Our primary outcome variable was pain during wound care as measured with FLACC. The results indicated a significant 40% reduction in pain during VR treatment compared to traditional treatment, as shown in Figure 5. Nurses observed that the children had significantly lower anxiety during VR treatment compared to traditional treatment (34% lower during VR; see Figure 6). Finally, comparable wound care treatment required significantly less time during VR (mean = 9.89 min during wound care with VR vs. 12.33 min for no VR, *p* < 0.01). 

Parents rated their child’s pain during burn wound care as well. As shown in Table 4, based on the parents’ observations of their children during wound care, parents reported that their children showed significant reductions in pain and anxiety during VR using single-item graphic ratings scales.

### Joy Scale Results

A sign test was used to measure the differences in children’s joy during VR treatments vs. during traditional treatment (no VR). One child self-reported their joy, while the parents assessed the joy of the other six patients. Note that data were missing for this question for two patients. Joy was significantly higher during adjunctive VR than during traditional treatment. The patients’ joy level during VR was rated as “pleased”, whereas joy during traditional treatment (No VR) was rated as “no joy”. The sign test results for the Joy Scale on a scale from zero to 5, where zero = no joy and 5 = ecstatic, showed a significant difference (no VR Joy Mean = 0.00, SD = 0.00 vs. Joy during VR = 2.29, SD = 1.13, *p* < 0.05).

As shown in Table 5, a post-study survey was distributed to participating doctors, nurses, and parents to obtain a preliminary assessment of the feasibility and acceptability of the desktop VR Animal Rescue intervention. The brief survey consisted of seven questions related to the VR experience. Twelve people participated in the survey. The respondents unanimously agreed that desktop Animal Rescue VR is feasible for use during burn wound care of children under six and is an intervention worth implementing. All of those surveyed indicated that VR did not delay wound care treatment. 

## 4. Discussion

The current study aimed to assess the effectiveness of a desktop VR distraction tool designed for young children under the age of 6 years as an adjunct to the current analgesic protocols typically used at these hospitals. Observational measures of pain and anxiety were used to evaluate children’s response before, during and after burn wound care procedures. Applying a desktop VR delivery platform is a novel means to make VR available to this young population, who would be unable to tolerate the application of a VR helmet. Burned children often have burns on their face/head, complicating the use of conventional head mounted VR goggles. Finding a way to improve the experience of young children during medical procedures is important, as most scald burns occur in children under 5 years old. At this stage of their development, children in this age group are more liable to explore their environments and are often oblivious to the dangers in their home environment, especially in the kitchen. Young children burn more quickly and at lower temperatures than older children and adults (American Burn Association (ABA), 2019) [46], and recent studies suggest that children under six are significantly more sensitive to certain types of pain stimuli than older children [55]. Researchers have recently begun to explore the use of “non-headset-delivered” VR for young children during burn wound care, e.g., rear-projection screens and interactive handheld devices [45,56].

The current study is part of a planned sequence of two related studies, one laboratory study [55] and the current clinical study. We first conducted a laboratory study with children aged 2–10 years old [55]. In that laboratory study, a within-subject repeated measures design was used. With the treatment order randomized, each healthy volunteer pediatric participant in our previous laboratory study underwent brief cutaneous pressure stimuli under three conditions: (1) no distraction, (2) a verbal color naming task (no VR), and (3) a large TV-based desktop VR distraction. A hand-held Wagner pressure pain stimulation device was used to generate just noticeable pain sensations. Participants indicated when a steadily increasing non-painful pressure stimulus first turned into a painful pressure sensation (i.e., just noticeable pain). One key result of our previous laboratory study using Animal Rescue World via desktop VR is the finding that children under 6 years old were significantly more sensitive to pain than children aged 6–10 during no distraction [55], and VR significantly reduced the “just noticeable” pressure pain sensitivity of children in both age groups. 

Although encouraging, the Alrimy et al. [55] laboratory study does not address the current issue of whether desktop VR can reduce pain during burn wound care. The participants in our previous laboratory study, Alrimy et al., 2022 [55], were healthy volunteers, whereas the participants in the current study were severely burned children. The duration of pressure pain in Alrimy et al., 2022 [55] was very brief; the painful stimuli consisted of barely painful “just noticeable pressure pain”, and each stimulus only lasted a few seconds. In contrast, the pain experienced by children during burn wound care in the current study lasted 10 min, and the intensity of pain during usual pain medications + No VR in the current study was severe pain, much higher intensity than in the laboratory study. Thus, the results of the present study are crucial for assessing the clinical value of desktop VR. Previous researchers have proposed that it may be difficult to distract people during severely painful procedures [57]. Whether young children would respond to desktop VR analgesia was unknown, and is addressed in the current study for the first time.

The average age of our patient population was 18 months. There was a significant improvement in analgesia and anxiolysis with our desktop VR program. No VR-related side effects were found, particularly related to vertigo, nausea, vomiting, or motion sickness. 

Limitations. Our study has a number of limitations. We used a within-subject repeated measures design, which is commonly employed in pilot studies. This design has an acknowledged limitation that the first treatment condition may potentially affect the results of the second treatment condition, i.e., there may be carry-over effects and bias artifacts [49]. Randomized controlled studies using more carefully controlled between groups designs, along with blinding and larger samples are recommended [58]. Another limitation is that most of the patients in our study were pre-verbal. The observational measures of pain (FLACC) and anxiety (PBCL) are commonly used and accepted in this very young age group; however, as with any observational scale, there is a risk of subjectivity on the part of the rater. 

Future Directions. In our post-treatment feasibility survey, all of the twelve doctors, nurses, and parents surveyed agreed that desktop VR with Animal Rescue World is feasible for use during burn wound care of children under six and is an intervention worth implementing. The results of the current study suggest that desktop VR is effective at reducing pain and anxiety for burn dressing procedures. Using desktop VR is a cost-effective means of delivering interactive VR; affordability is especially important in this context, as the majority of young children who suffer extreme burns are from under-developed countries, and children from all countries are often undermedicated. Further large-scale studies are needed to explore the clinical applications of Animal World VR in infants and younger age group. Studies exploring long term benefits of VR are especially needed due to benefits including, e.g., reduced risk of children developing costly PTSD and chronic pain. By reducing aversive conditioning, VR may reduce children’s risk of developing avoidance of healthcare. Whether desktop VR can reduce the pain and anxiety of children under six during venipuncture is another important topic [59], as venipuncture is one of the most frequent procedures in medicine [60].

Future studies might explore whether desktop VR programs such as Animal Rescue World can help to reduce pre-surgical anxiety. Young children needing MRI scans could play Animal Rescue World in the MRI tube by using common prismatic glasses to see the computer screen at the top opening of the tube, and could use a magnet-friendly mouse to interact with the virtual animals during their MRI scan. Future studies might additionally explore playing Animal Rescue World using immersive VR, augmented reality [61,62] via head-mounted displays, or desktop VR to help distract older children during venipuncture and dental procedures. Because it was built using the Unity software architecture, Animal Rescue World can be used with immersive VR in contexts where wearing a VR helmet is appropriate or with desktop VR. Most importantly, desktop VR opens up the use of VR for children under six and children who cannot wear a VR helmet, e.g., when using VR as a non-pharmacologic sedation during MRI scans.

## 5. Conclusions

In the current study, the patient population was children under six with severe burns requiring burn wound care in a hospital, e.g., scald burns. Nurse’s ratings of patient’s pain behaviors during wound care with and without VR was the primary outcome measure. During burn wound care with no distraction (traditional pain medications), children under 6 years old experienced severe pain during approximately 10 minutes of burn wound cleaning. When combining desktop virtual reality + traditional pain medication during burn wound care, the same patients experienced only mild pain during burn wound cleaning/debridement. VR significantly reduced the children’s pain during burn wound care. The encouraging preliminary results from the current pilot study indicate that additional research and development of VR for children under six is warranted.

## Figures and Tables

**Table 1:** Demographic characteristics of the participants.

		Frequency			Percent		
**Gender**	Male	3			33.0%		
	Female	6			67.0%		
**Hospitalized**	Yes	8			89%		
	No (Clinic)	1			11%		
**Cause of Injury**	Thermal (Scaled)						
	Burn	9			100.0%		
**Hospital**	King Abdulaziz	4			44.5%		
	Alnoor	4			44.5%		
	IMC	1			11.0%		
**Background Medication**	Paracetamol	9			100.0%		
**Total**		9			100.0%		
	**Minimum**	**Maximum**		**Mean**		**Std. D**	
**Age in months 10**	10	60		18.89		15.90	
**Weight**	8	16		10.31		2.39	
**Burn extent**	3%	22%		10%		6%	
**Previous wound care sessions**	1	15		4.33		4.18	

**Table 2:** Injury/burn characteristics of the participants.

Injury/Burn Type	Frequency	Percent
*Deep partial thickness (second degree)*	2	22.2%
*Superficial (first degree), Superficial thickness (second degree)*	1	11.1%
*Superficial thickness (second degree)*	3	33.3%
*Superficial thickness (second degree), Full thickness (third degree)*	1	11.1%
*Superficial thickness (second degree), deep thickness (second degree)*	2	22.2%
**Total**	**9**	**100%**
**Areas of Burn**	**Frequency**	**Percent**
*anterior trunk, right legs, right posterior trunk, right hand*	*1*	*11.1%*
*Both Legs*	*1*	*11.1%*
*chest*	*1*	*11.1%*
*Face & Chest*	*2*	*22.2%*
*left leg and hand*	*1*	*11.1%*
*Right hand, left leg*	*1*	*11.1%*
*right side of face, right upper limb and truck, hand*	*1*	*11.1%*
*thigh, both legs (anterior aspect), genitalia*	*1*	*11.1%*
**Total**	**9**	**100%**

**Table 3:** The paired sign test results for nurse’s ratings of the child’s pain and anxiety during wound care.

	Group	N	Mean (Std. D)	Two Tailed *p*-Value
Pain Before Wound care	VR	9	2.11 (SD = 1.69)	*p* > 0.05 NS
	Traditional	9	2.89 (SD = 2.32)	
**Pain During Wound care**	**VR**	**9**	**4.00 (SD = 2.24)**	***p*** < 0.01 **
	**Traditional**	**9**	**6.67 (SD = 2.45)**	
Pain After Wound care	VR	9	1.56 (1.13)	*p* < 0.05 *
	Traditional	9	3.33 (1.73)	
Anxiety Before Wound care	VR	9	6.44 (SD = 7.30)	Z = 1.19 *p* > 0.05 NS
	Traditional	9	7.33 (SD = 7.62)	
Anxiety During Wound care	VR	9	13.67 (SD = 8.93)	Z = 2.67, *p* < 0.01 **
	Traditional	9	20.56 (SD = 8.58)	
Anxiety After Wound care	VR	9	2.33 (SD = 1.80)	Z = 2.25, *p* < 0.05 *
	Traditional	9	6.11 (SD = 4.70)	

* significance at *p* < 0.05 level; ** significance at *p* < 0.01 level; NS = Non-Significant.

**Table 4:** The sign test results for parents’ ratings of their child’s pain and anxiety during wound care.

	Group	N	Mean (Std. D)	Two Tailed *p*-Value
Pain Before Wound care	VR	9	1.89 (1.17)	*p* > 0.05 NS
	Traditional	9	2.11 (1.05)	
**Pain During Wound care**	**VR**	**9**	**2.56 (1.01)**	***p*** < 0.05 *
	**Traditional**	**9**	**4.00 (1.12)**	
Pain after Wound Care	VR	9	1.67 (0.87)	*p* > 0.05 NS
	Traditional	9	2.56 (1.33)	
Anxiety Before Wound care	VR	9	2.67 (1.73)	*p* > 0.05 NS
	Traditional	9	2.78 (1.92)	
Anxiety During Wound care	VR	9	2.89 (1.36)	*p* < 0.01 **
	Traditional	9	4.67 (0.71)	
Anxiety After Wound care	VR	9	1.56 (0.88)	*p* < 0.05 *
	Traditional	9	3.11 (1.45)	

* significance at *p* < 0.05 level; ** significance at *p* < 0.01 level; NS = Non-Significant.

**Table 5:** Results of the post-study survey.

Question 1	Virtual Reality helped the child control his/her pain.	100% responded either total agree or agree (33% total agree, 67% agree).
Question 2	VR helped the child to cooperate during the medical procedure	100% responded either total agree or agree (42% total agree, 52% agree).
Question 3	Use of VR delayed the wound care process related to the procedure	100% responded either totally disagree, or disagree (17% totally disagree, 83% disagree).
Question 4	I would use VR again to distract children during a painful procedure	100% responded either totally agree or agree (75% totally agree, 25% agree).
Question 5	The VR game was adapted/suitable to the age group of children	100% responded either totally agree or agree (62% totally agree, 38% agree).
Question 6	The VR device was adapted/suitable to the clinic’s environment	100% responded either totally agree or agree (67% totally agree, 33% agree).
Question 7	VR is an intervention worth implementing to distract children	100% responded either totally agree or agree (75% totally agree, 25% agree).

## Data Availability

Please contact TA with requests for additional data summaries (e.g., for meta-analyses).
